# Aliskiren attenuates cardiac dysfunction by modulation of the mTOR and apoptosis pathways

**DOI:** 10.1590/1414-431X20198793

**Published:** 2020-01-24

**Authors:** Zhengbo Zhao, Han Liu, Dongmei Guo

**Affiliations:** 1 Department of Cardiovascular Medicine, Jiulongpo District People's Hospital, Chongqing, China; 2 Department of Neurology, Jiulongpo District People's Hospital, Chongqing, China; 3 Department of Cardiovascular Medicine, Nanchuan District People's Hospital, Chongqing, China

**Keywords:** Aliskiren, Cardiac hypertrophy, Fibrosis, Mammalian target of rapamycin, Apoptosis

## Abstract

Aliskiren (ALS) is well known for its antihypertensive properties. However, the potential underlying the molecular mechanism and the anti-hypertrophic effect of ALS have not yet been fully elucidated. The aim of the present study was to investigate the role of ALS in mammalian target of rapamycin (mTOR) and apoptosis signaling using *in vivo* and *in vitro* models of cardiac hypertrophy. A rat model of cardiac hypertrophy was induced by isoproterenol treatment (5 mg·kg^-1^·day^-1^) for 4 weeks, with or without ALS treatment at 20 mg·kg^-1^·day^-1^. The expression of hypertrophic, fibrotic, and apoptotic markers was determined by RT-qPCR. The protein expression of apoptotic markers mTOR and p-mTOR was assessed by western blot analysis. The proliferation of H9C2 cells was monitored using the MTS assay. Cell apoptosis was analyzed using flow cytometry. *In vivo*, isoproterenol-treated rats exhibited worse cardiac function, whereas ALS treatment reversed these dysfunctions, which were associated with changes in p-mTOR, Bcl-2, Bax, and cleaved caspase-3 expression, as well as the number of apoptotic cells. *In vitro*, H9C2 cardiomyocyte viability was significantly inhibited and cardiac hypertrophy was induced by Ang II administration, but ALS reversed Ang II-induced H9C2 cardiomyocyte hypertrophy and death. Furthermore, Ang II triggered the activation of the mTOR and apoptosis pathways in hypertrophic cardiomyocytes that were inhibited by ALS treatment. These results indicated that ALS alleviated cardiac hypertrophy through inhibition of the mTOR and apoptosis pathways in cardiomyocytes.

## Introduction

Cardiac hypertrophy is one of the main causes of cardiovascular morbidity and mortality, and its prevention may represent a new management strategy for improving survival in patients with cardiovascular diseases (CVDs) (1). The pathological mechanisms of cardiac hypertrophy involve various molecular and signaling pathways, including those associated with oxidative stress (2), autophagy (3), and inflammatory response (4). The mammalian target of rapamycin (mTOR) signaling cascade is implicated in the induction of cardiac hypertrophy and fibrosis (5). Several *in vivo* and *in vitro* studies have documented that the mTOR pathway is a promising therapeutic target for pressure overload-, isoproterenol (ISO)- and angiotensin (Ang) II-induced cardiac hypertrophy, and fibrosis ([Bibr B06] [Bibr B07] 68). However, Song et al. demonstrated that mTOR attenuates pressure overload-induced cardiac dysfunction and hypertrophy by regulation of the inflammatory reaction (9). Kemi et al. (10) demonstrated that pressure overload is associated with inactivation of the mTOR pathway. These findings suggest that the role of mTOR in the pathological process of cardiac hypertrophy and fibrosis has not been fully defined.

The renin-angiotensin system (RAS) and Ang II, a key active peptide in RAS, have been shown to be closely associated with cardiac hypertrophy and fibrosis, which may be prevented by angiotensin-converting enzyme inhibitor (ACEI) or Ang II type 1 receptor blocker (ARB) treatment (11,12). However, ACEI and ARB only partially protect against the progression of CVDs, which may be attributed to the increase in plasma renin activity and the production of Ang I (13). This phenomenon may lead to ‘ACE escape' and restore the circulating Ang II to baseline levels in patients receiving long-term treatment with ACEI or ARB (14). Aliskiren (ALS) is a renin inhibitor that prevents the formation of Ang I from angiotensinogen, and may represent a novel approach to restricting RAS by directly inhibiting this system at its rate-limiting proximal step (15). Previous studies reported that ALS is as effective as ACEI or ARB in controlling blood pressure (1,16,17). Recently, the antihypertrophic and antifibrotic effects of ALS have been emerging in experimental and clinical studies (1,14,18). However, the mechanisms underlying the therapeutic effects of ALS on cardiac hypertrophy and fibrosis remain poorly understood.

The aim of the present study was to investigate whether ALS protects against ISO-induced cardiac hypertrophy and fibrosis in a rat model and Ang II-induced cardiac hypertrophy *in vitro*. The apoptotic mechanism and AKT/mTOR pathway, which may contribute to the antihypertrophic and antifibrotic effects of ALS *in vivo* and *in vitro*, were also investigated.

## Material and Methods

### Animal treatment

A total of 24 ten-week-old male Sprague-Dawley rats (body weight 200–250 g) were purchased from Vital River Laboratories Co., Ltd. (China) and allowed to acclimatize to the environment for 1 week. The rats were randomly divided into four groups: Vehicle group rats (n=6) received normal saline by subcutaneous injection; ISO group rats (n=6) were treated with ISO (Santa Cruz Biotechnology, USA) at a dose of 5 mg/kg per day by subcutaneous injection for 2 weeks to induce cardiac hypertrophy as described previously (19,20); ALS group rats (n=6) were treated with ALS (cat. No. CDS023114; purity: ≥98%; Sigma-Aldrich; Merck KGaA, USA; formula: C30H53N3O6) at a dose of 20 mg/kg per day by intragastric administration; and ALS+ISO group rats (n=6) were treated with ISO (5 mg/kg per day) combined with ALS (20 mg·kg^-1^·day^-1^). All the rats were sacrificed by an overdose of sodium pentobarbital (2%; 200 mg/kg; Sigma-Aldrich; Merck KGaA) after 4 weeks of treatment. The hearts were collected and immediately weighed, and then frozen in liquid nitrogen for gene and protein analysis, or fixed in 4% formalin at room temperature and embedded in paraffin for histological analysis. The experiments were approved by the Ethics Committee of the Nanchuan District People's Hospital (China).

### Hematoxylin and eosin (H&E) staining

The left ventricles (LVs) were collected and fixed with 4% formalin at room temperature for 24 h, and then embedded in paraffin. Tissues were cut into 3-μm sections, which were stained with H&E (Beyotime Institute of Biotechnology, China) at room temperature and visualized under a microscope (Leica DM 2500; Leica Microsystems GmbH, Germany). The width of cardiomyocytes was measured with an automated image analysis system (Image-Pro Plus 5.0, Media Cybernetics, USA).

### RNA extraction and reverse transcription-quantitative polymerase chain reaction (RT-qPCR) analysis

Total RNA was extracted using TRIzol^®^ (Invitrogen; Thermo Fisher Scientific, Inc., USA), according to the manufacturer's protocol. cDNA was synthesized by reverse transcription reactions with 2 μg total RNA using Moloney Murine Leukemia Virus Reverse Transcriptase (Invitrogen; Thermo Fisher Scientific, Inc.). PCR analysis was performed using the TaqMan Universal PCR Master Mix (Thermo Fisher Scientific, Inc.) with a DNA Engine (ABI 7300; Thermo Fisher Scientific, Inc.). The reaction conditions were as recommended by the manufacturer's protocol as follows: 95°C for 10 min, 35 cycles of 95°C for 15 s, 60°C for 30 s, and 72°C for 30 s. The PCR primers are shown in Table 1. Glyceraldehyde-3-phosphate dehydrogenase (GAPDH) levels were used to normalize the expression of the target genes. The relative gene expression levels were calculated using the 2^-ΔΔCq^ method (21).

**Table 1:** Primers used in the RT-qPCR.

Gene	Forward primer (5′-3′)	Reverse primer (5′-3′)
ANP	GGAGCCTGCGAAGGTCAA	TATCTTCGGTACCGGAAGCTGT
BNP	CAGAAGCTGCTGGAGCTGATAAG	TGTAGGGCCTTGGTCCTTTG
α-MHC	GCCCTTTGACATCCGCACAGAGT	TCTGCTGCATCACCTGGTCCTCC
β-MHC	GCGGACATTGCCGAGTCCCAG	GCTCCAGGTCTCAGGGCTTCACA
Procollagen I	TATGCTTGATCTGTATCTGCCACAAT	TCGCCCTCCCGTTTTTG
Procollagen III	CAGCTGGCCTTCCTCAGACT	TGCTGTTTTTGCAGTGGTATGTAA
Bcl-2	GAGCGTCAACAGGGAGATGT	CAGCCAGGAGAAATCAAACAG
Bax	TTGCTACAGGGTTTCATCCA	TGTTGTTGTCCAGTTCATCG
Caspase3	AGCTGGACTGCGGTATTGAG	AGCTGGACTGCGGTATTGAG
GAPDH	GCACCGTCAAGCTGAGAAC	TGGTGAAGACGCCAGTGGA

### Western blotting

Protein was extracted using RIPA Lysis buffer (Beyotime Institute of Biotechnology, China). The concentration was determined using the Bicinchoninic Acid Kit for Protein Determination (Sigma-Aldrich; Merck KGaA). Samples containing 30 μg protein were separated by 10% SDS-PAGE and transferred onto nitrocellulose membranes (Bio-Rad Laboratories, Inc., USA). Primary antibodies against mTOR (cat. No. sc-293089, dilution, 1:1000), p-mTOR (cat. No. sc-293132, dilution, 1:500), Bcl-2 (cat. No. sc-56015, dilution, 1:1000) and Bax (cat. No. sc-6236, dilution, 1:1000) were purchased from Santa Cruz Biotechnology. Cleaved caspase-3 (cat. No. 9661, dilution, 1:1000) was purchased from Cell Signaling Technology, Inc. (USA). After incubation with primary antibodies at room temperature for 2 h, the membranes were incubated with the appropriate horseradish peroxidase-conjugated secondary antibody (cat. No. sc-516102; dilution, 1:10,000; Santa Cruz Biotechnology), following visualization using chemiluminescence (Thermo Fisher Scientific, Inc.). β-actin (cat. No. sc-130301; dilution, 1:2000; Santa Cruz Biotechnology) was used as the control antibody. Signals were analyzed with Quantity One^®^ software, version 4.5 (Bio-Rad Laboratories, Inc.).

### Cell culture

H9C2 cells were purchased from the American Type Culture Collection (Bethesda, USA) and cultured in Dulbecco's modified Eagle's medium (DMEM; Gibco; Thermo Fisher Scientific, Inc., USA) containing 10% fetal calf serum (Gibco; Thermo Fisher Scientific, Inc.), 10% L-glutamine, 0.5% penicillin/streptomycin, 10% non-essential amino acids, and 10% pyruvate in a 5% CO_2_ atmosphere at 37°C. H9C2 cells were treated with Ang II alone (10 μM, Sigma-Aldrich; Merck KGaA) or combined with ALS (20 μM) or rapamycin (RAP; 100 nM, Sigma-Aldrich; Merck KGaA). All the experiments were performed in triplicate.

### MTS assay

The proliferation of H9C2 cells was monitored using the MTS assay kit (Promega Corporation, USA). Absorbance was measured at 492 nm using an ELISA reader (MD SpectraMax M5; Molecular Devices, LLC, USA). The MTS assay was performed as described previously (22).

### Flow cytometry analysis

H9C2 cells were treated with Ang II (10 μM), Ang II (10 μM) + ALS (20 μM), or Ang II (10 μM) + RAP (100 nM) for 24 h. Cells were collected after digestion, washed twice with PBS, and centrifuged at 1,500 *g* for 5 min at 4°C. The supernatant was discarded, and the cells were resuspended, fixed in ice-cold 75% ethanol, and stored at 4°C. The cell apoptosis assay was conducted as previously described (23). The Annexin V-FITC apoptosis detection kit was purchased from Invitrogen (Thermo Fisher Scientific). The samples were analyzed using a flow cytometer (BD Biosciences, USA). The data were processed by Cell Quest Software (version 5.1, BD Biosciences).

### Statistical analysis

Data are reported as means±SE. Statistical analysis was performed using GraphPad Prism version 7.0 (GraphPad Software, Inc., USA). Inter-group differences were analyzed by one-way analysis of variance, followed by Tukey's *post hoc* analysis. P<0.05 was considered to be a statistically significant difference.

## Results

### ALS attenuated ISO-induced cardiac hypertrophy and fibrosis in rats

**Figure 1:**
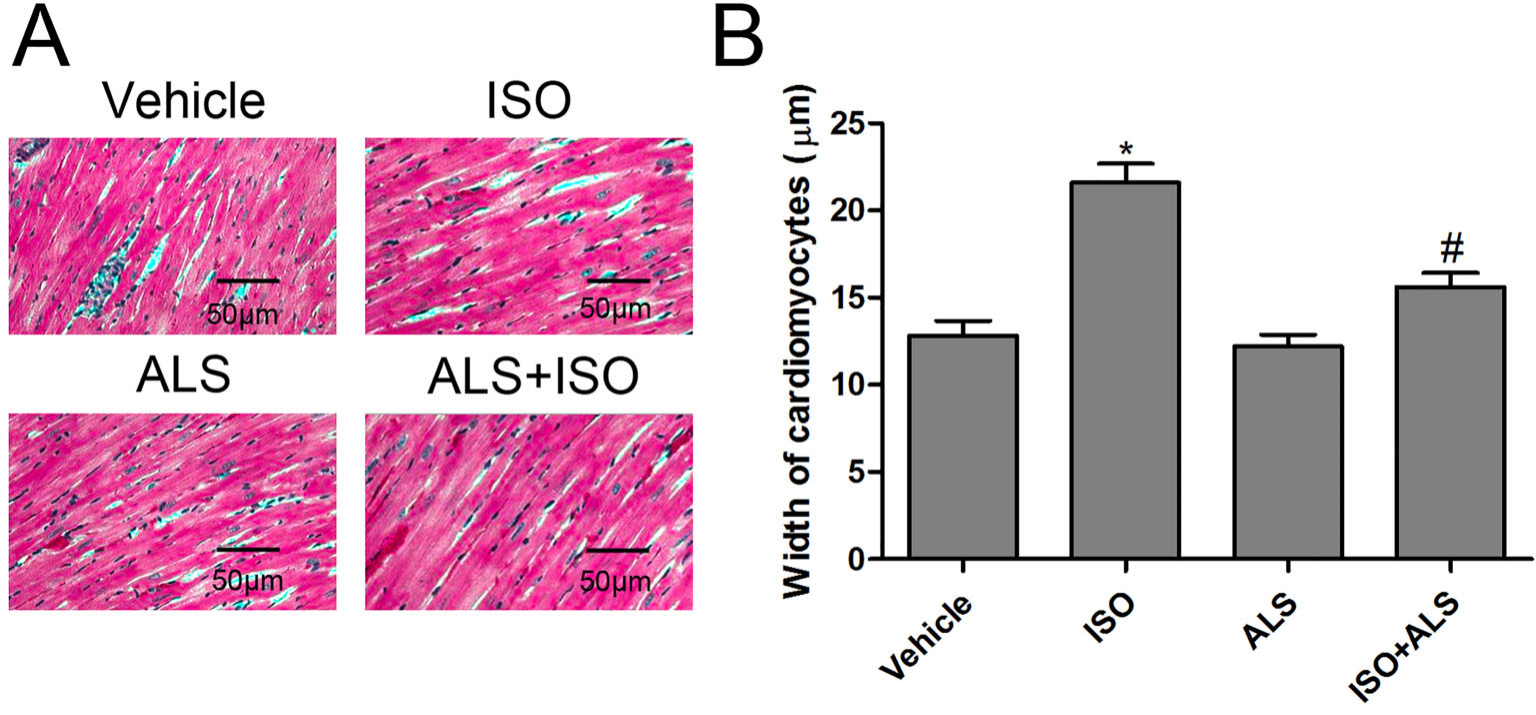
Effect of aliskiren (ALS) on cardiomyocyte size in isoproterenol (ISO)-treated rats. **A**, Hematoxylin and eosin staining was performed and (**B**) the width of cardiomyocytes was measured (n=6 per group). Data are reported as means±SE. *P<0.05 compared with vehicle; ^#^P<0.05 compared with the ISO-treated group (ANOVA followed by Tukey's *post hoc* test).

**Figure 2:**
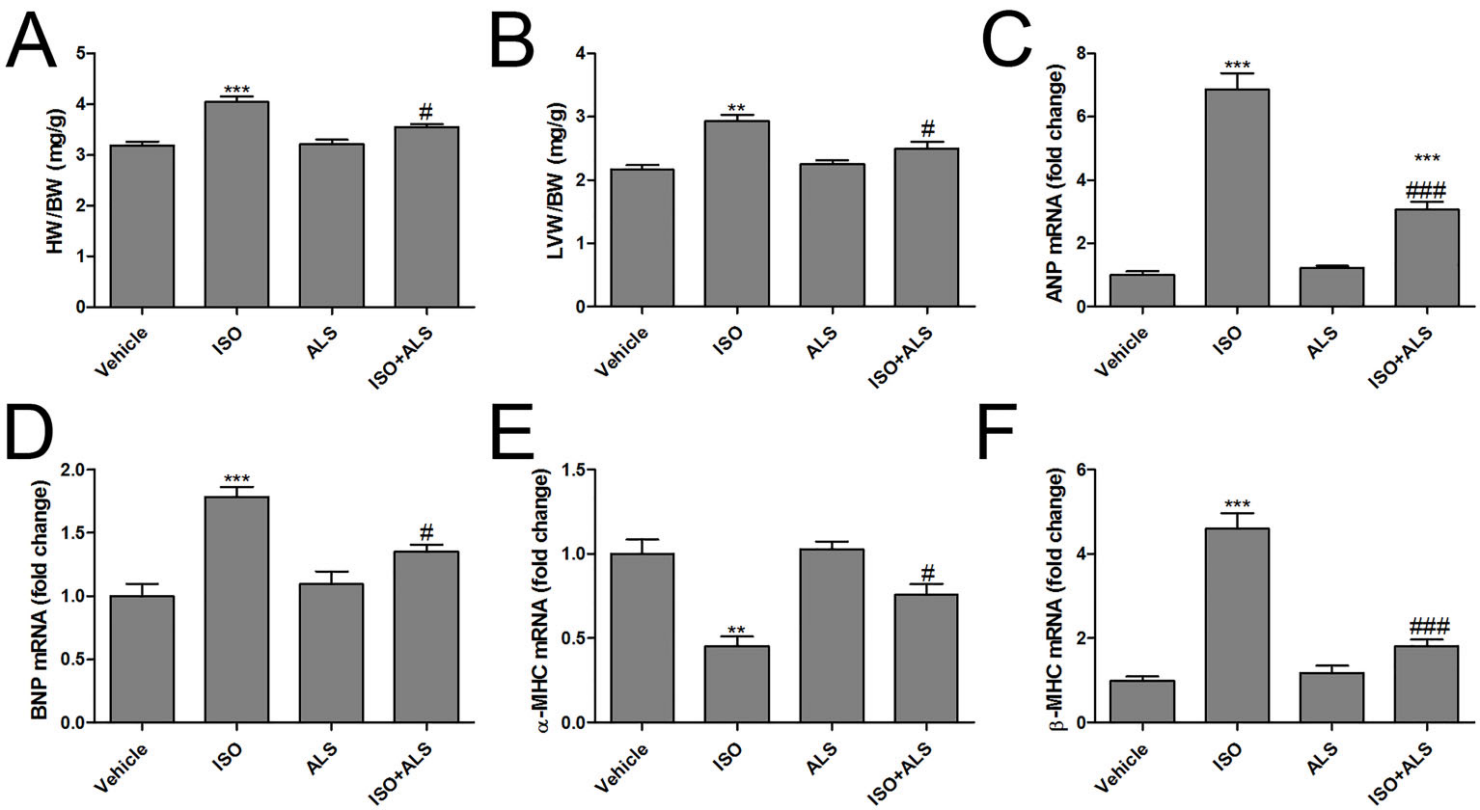
Canonical markers of cardiac hypertrophy in response to aliskiren (ALS) in isoproterenol (ISO)-treated rats. The ratio of (**A**) heart weight/body weight (HW/BW) and (**B**) left ventricular weight to body weight (LVW/BW), the mRNA levels of (**C**) atrial natriuretic peptide (ANP), (**D**) brain natriuretic peptide (BNP), (**E**) α-myosin heavy chain (α-MHC), and (**F**) β-MHC were measured in the left ventricles of the rats (n=6 per group). Data are reported as means±SE. **P<0.01, ***P<0.001 compared with vehicle; ^#^P<0.05, ^###^P<0.001 compared with the ISO group (ANOVA followed by Tukey's *post hoc* test).

To investigate the cardioprotective effects of ALS on ISO-induced cardiac hypertrophy and fibrosis *in vivo*, a rat model of cardiac hypertrophy and fibrosis was successfully constructed. First, it was observed that ISO induced marked cardiac hypertrophy by increasing the cardiomyocyte width, which was significantly suppressed in the ALS+ISO treatment group (Figure 1A and B). In addition, both the ratio of heart weight to body weight (HW/BW) and LV weight to BW (LVW/BW) were increased following ISO injection, but HW/BW and LVW/BW were lowered in the combined ALS + ISO group (Figure 2A and B). Furthermore, the hypertrophic markers, including atrial natriuretic peptide (ANP) and brain natriuretic peptide (BNP), α-myosin heavy chain (α-MHC) and β-myosin heavy chain (β-MHC), were evaluated in the LVs of rats. RT-qPCR analysis demonstrated that the levels of ANP, BNP, and β-MHC were significantly higher in the LVs from ISO-treated rats compared with the control group, but treatment with ALS significantly decreased the ISO-mediated increase in ANP, BNP, and β-MHC mRNA expression (Figure 2C, D and F). It was also observed that ISO treatment markedly decreased α-MHC mRNA expression. However, ALS treatment significantly reversed the ISO-induced downregulation of α-MHC in the LVs of rats (Figure 2E).

### Effect of ALS on fibrotic markers associated with cardiac hypertrophy

**Figure 3:**
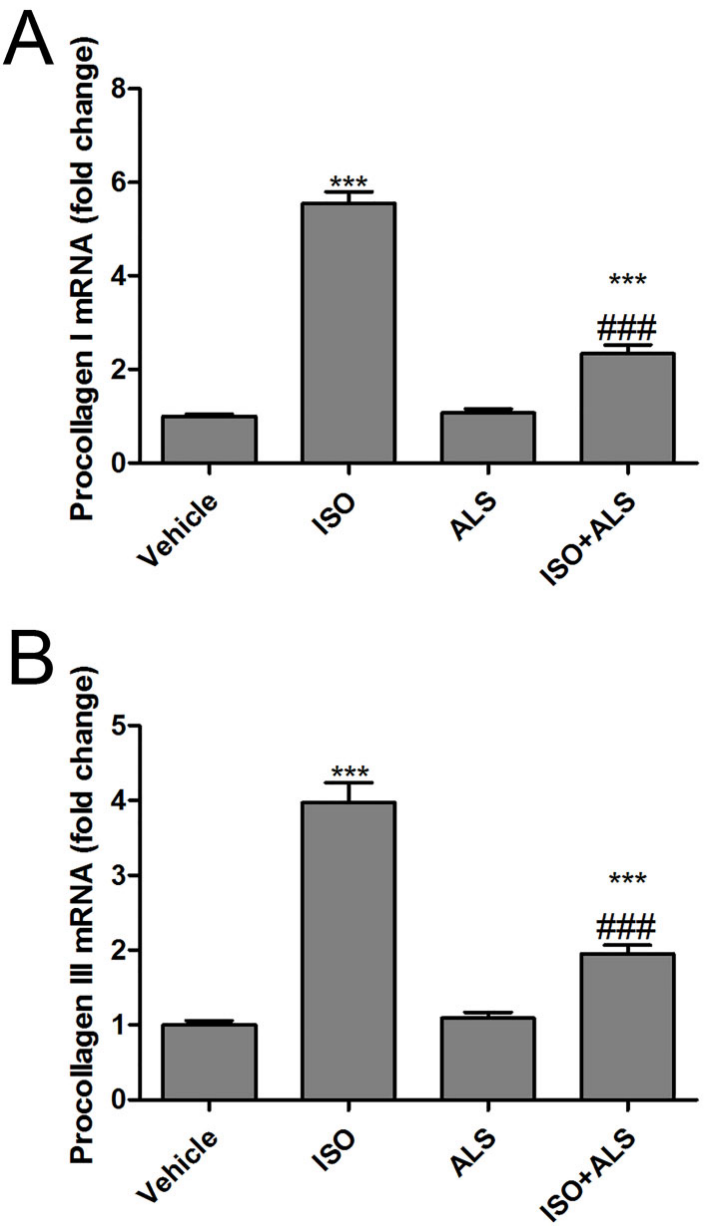
Canonical markers of cardiac fibrosis in response to aliskiren (ALS) in isoproterenol (ISO)-treated rats. The mRNA expression of (**A**) procollagen I and (**B**) procollagen III was measured by RT-qPCR in the left ventricles of the rats (n=6 per group). Data are reported as means±SE. ***P<0.001 compared with vehicle; ^###^P<0.001 compared with the ISO group (ANOVA followed by Tukey's *post hoc* test).

To investigate the effect of ALS on fibrosis in ISO-treated rats, the cardiac gene expression of the fibrotic markers procollagen I and III was measured. The results demonstrated that ISO treatment significantly upregulated procollagen I and III mRNA expression compared with the control group, whereas ALS suppressed the fibrotic responses in LVs triggered by ISO (Figure 3A and B).

### Inhibition of apoptosis and mTOR signaling by ALS in ISO-treated rats

**Figure 4:**
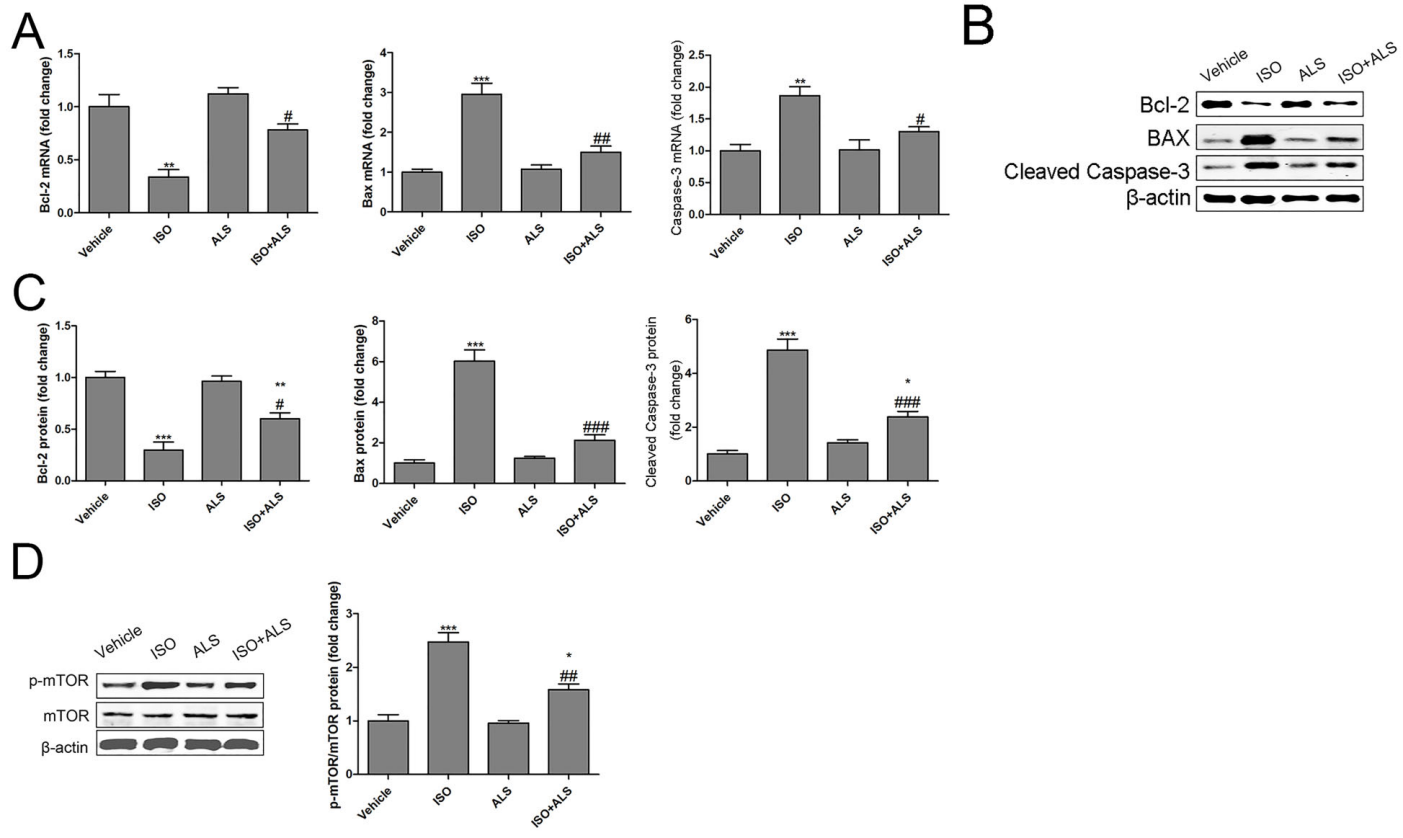
Aliskiren (ALS) inhibited the mTOR and apoptosis pathways in isoproterenol (ISO)-treated rats. The mRNA expression of Bcl-2, Bax, and caspase-3 was measured by RT-qPCR in the left ventricles of the rats (n=6 per group) (**A**). The protein expression of Bcl-2, Bax, and cleaved caspase-3 was measured by western blotting in the left ventricles of the rats (**B** and **C**). The protein expression of mTOR and p-mTOR was measured by western blotting in the left ventricles of the rats (**D**) (n=6 per group). Data are reported as means±SE. *P<0.05, **P<0.01, ***P<0.001 compared with vehicle; ^#^P<0.05, ^##^P<0.01, ^###^P<0.001 compared with the ISO group (ANOVA followed by Tukey's *post hoc* test). mTOR: mammalian target of rapamycin.

To determine the molecular mechanisms of ALS in ISO-induced cardiac hypertrophy and fibrosis, the apoptosis and mTOR pathways were assessed by RT-qPCR and western blotting in isolated rat LVs. The apoptosis-related markers Bax and caspase-3, and the anti-apoptosis protein Bcl-2 were evaluated. ISO increased the mRNA and protein expression of Bax and caspase-3, and suppressed the mRNA and protein expression of Bcl-2 in isolated LVs; these effects were blocked by ALS treatment (Figure 4A, B, and C). Moreover, the phosphorylation levels of the mTOR protein exhibited a significant increase in the ISO-treated group. However, ALS treatment effectively inhibited the activation of the mTOR signaling pathway triggered by ISO (Figure 4D).

### ALS prevented Ang II-modulated apoptosis and mTOR signaling

**Figure 5:**
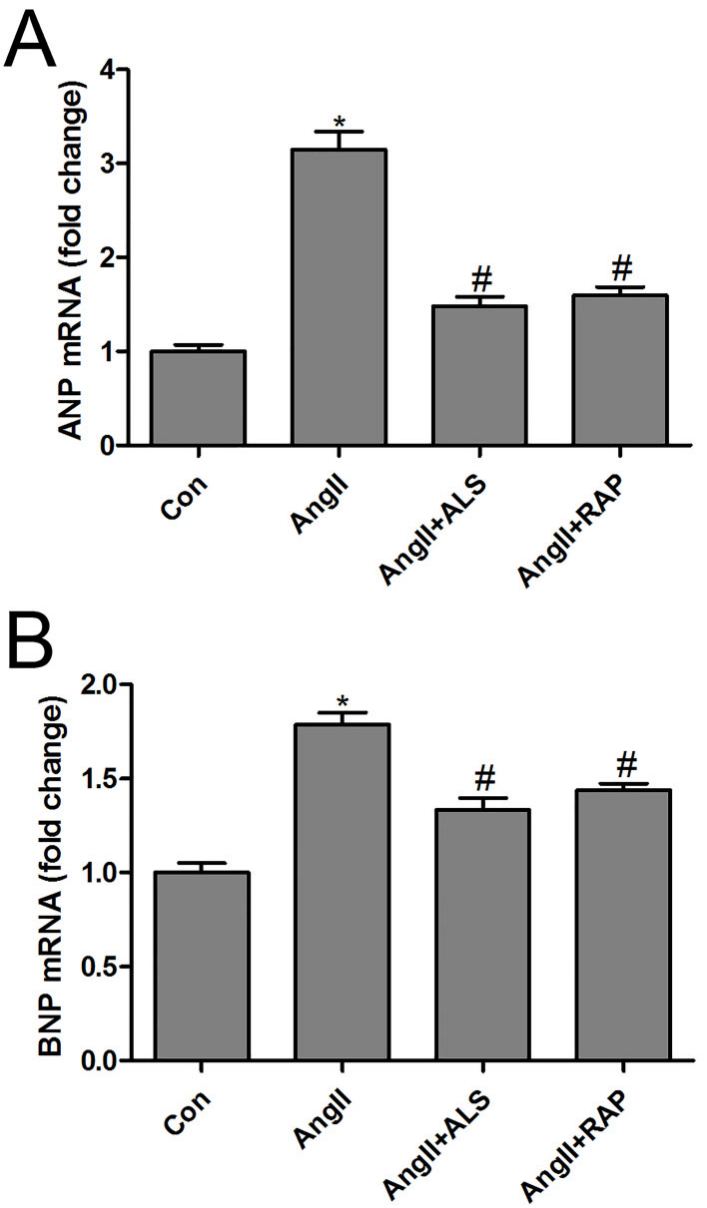
Aliskiren (ALS) decreased the markers of cardiac hypertrophy in Ang II-treated H9C2 cardiomyocytes. The mRNA expression of (**A**) ANP and (**B**) BNP was measured by RT-qPCR in the H9C2 cardiomyocytes after treatment with Ang II (10 μM), Ang II (10 μM) + ALS (20 μM), or Ang II (10 μM) + RAP (100 nM) for 48 h (n=3 per group). Data are reported as means±SE. *P<0.05 compared with control; ^#^P<0.05 compared with the Ang II-treated group (ANOVA followed by Tukey's *post hoc* test). Ang: angiotensin; ANP: atrial natriuretic peptide; BNP: brain natriuretic peptide; RT-qPCR: reverse transcription-quantitative polymerase chain reaction.

**Figure 6:**
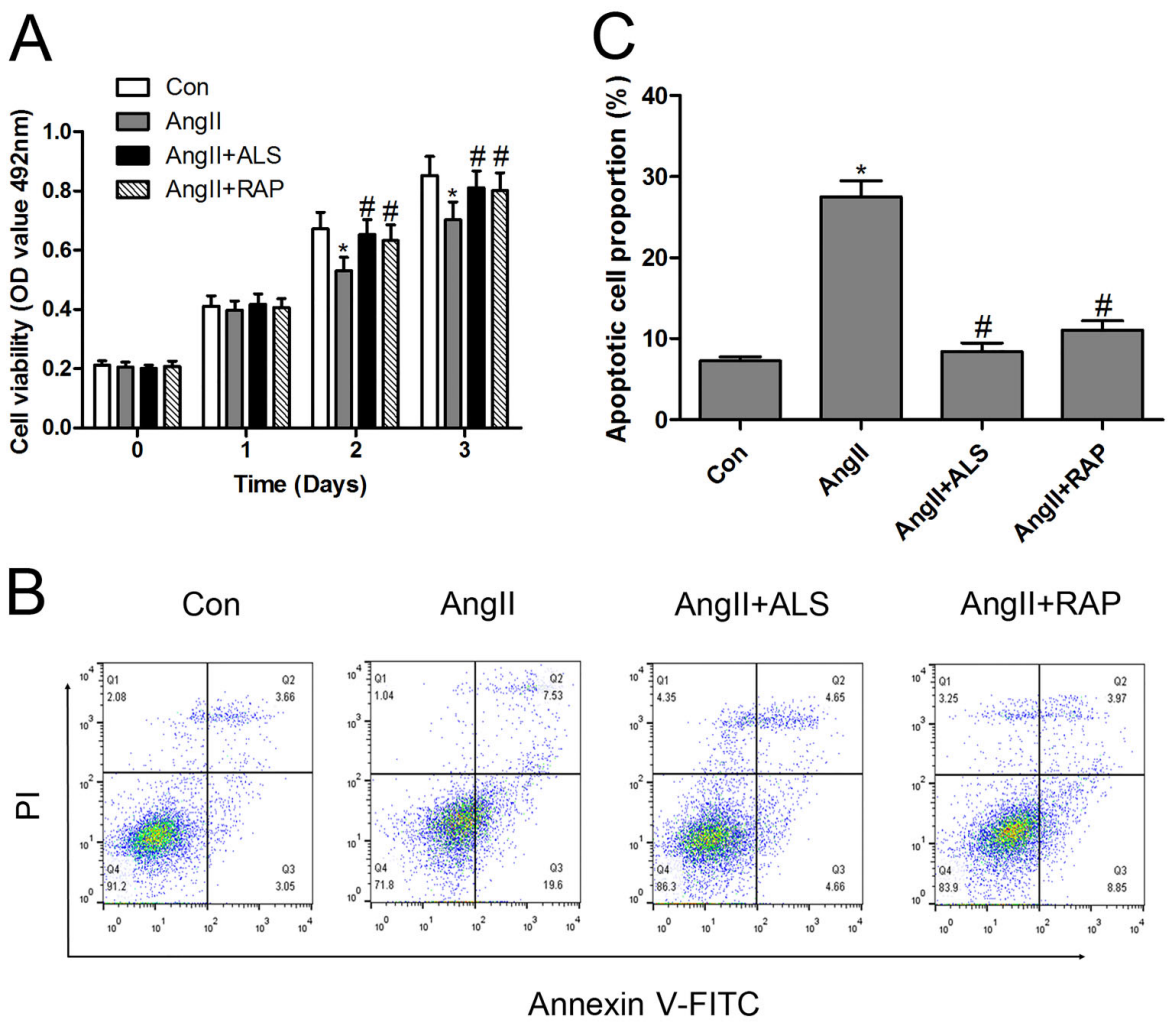
Aliskiren (ALS) improved cell viability and inhibited apoptosis in Ang II-treated H9C2 cardiomyocytes. After H9C2 cardiomyocytes were treated with Ang II (10 μM), Ang II (10 μM) + ALS (20 μM), or Ang II (10 μM) + RAP (100 nM), (**A**) the cell viability was monitored using MTS assay at 24, 48, and 72 h, and (**B** and **C**) apoptosis was analyzed using flow cytometry at 48 h (n=3 per group). Data are reported as means±SE. *P<0.05 compared with control; ^#^P<0.05 compared with the Ang II-treated group (ANOVA followed by Tukey's *post hoc* test). Ang: angiotensin; RAP: rapamycin.

**Figure 7:**
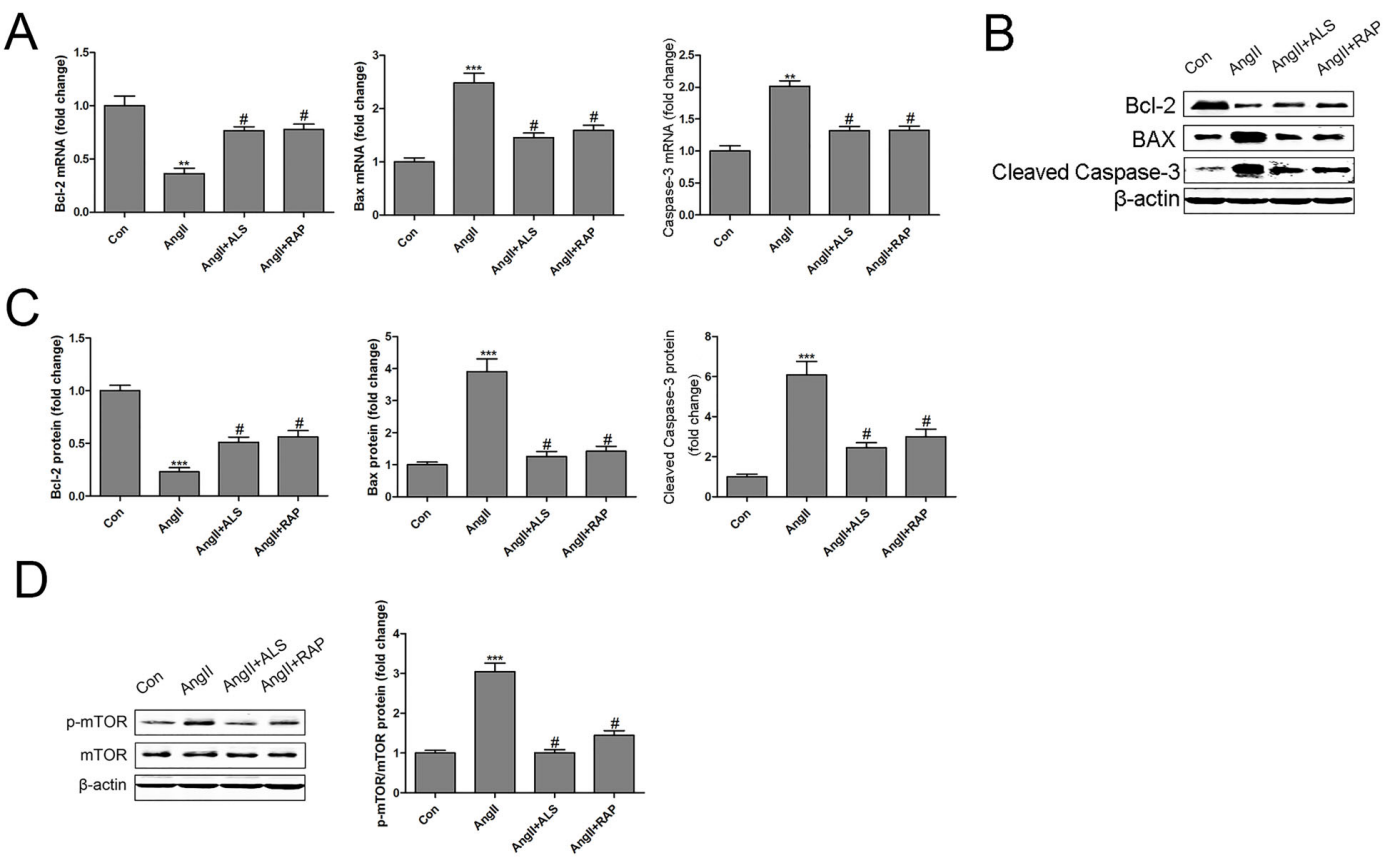
Aliskiren (ALS) inhibited the mTOR and apoptosis pathways in Ang II-treated H9C2 cardiomyocytes. The mRNA expression of Bcl-2, Bax, and caspase-3 was measured by RT-qPCR in H9C2 cardiomyocytes after treatment with Ang II (10 μM), Ang II (10 μM) + ALS (20 μM), or Ang II (10 μM) + RAP (100 nM) for 48 h (n=3 per group) (**A**). The protein expression of Bcl-2, Bax, and cleaved caspase-3 was measured by western blotting (**B** and **C**). The protein expression of mTOR and p-mTOR was measured by western blotting in H9C2 cardiomyocytes after treatment with Ang II (10 μM), Ang II (10 μM) + ALS (20 μM) or Ang II (10 μM) + RAP (100 nM) for 48 h (**D**), n=3 per group. Data are reported as means±SE. **P<0.01, ***P<0.001 compared with control; ^#^P<0.05 compared with the Ang II-treated group (ANOVA followed by Tukey's *post hoc* test). mTOR: mammalian target of rapamycin; Ang: angiotensin; RAP: rapamycin.

First, the hypertrophy markers ANP and BNP were investigated in Ang II-treated H9C2 cardiomyocytes. The results suggested that Ang II treatment significantly upregulated the mRNA levels of ANP and BNP, suggesting that Ang II successfully induced hypertrophy in cardiomyocytes *in vitro* (Figure 5A and B). Next, the potential cytotoxicity of Ang II, ALS, and RAP was analyzed using an MTS assay. H9C2 cardiomyocytes were treated with Ang II, Ang II+ALS, or Ang II+RAP for different times. Cell viability did not differ notably at 24 h of incubation. However, cell viability was significantly inhibited by Ang II at 48 and 72 h. Both ALS and RAP inhibited Ang II-induced H9C2 cardiomyocyte death (Figure 6A). Similar results were obtained by flow cytometry analysis after treatment with different conditions at 48 h (Figure 6B and C). To confirm the modulatory effect of the apoptosis and mTOR pathways on the ALS-mediated cardioprotective effect against Ang II-induced cardiac hypertrophy *in vitro*, H9C2 cardiomyocytes were treated with Ang II, ALS, or RAP (an inhibitor of mTOR). The results indicated that both ALS and RAP inhibited Ang II-activated apoptosis and mTOR signaling *in vitro* (Figure 7A-D), which were consistent with the results *in vivo*. These data provided evidence that ALS treatment improved cardiac hypertrophy and fibrosis by inhibiting the mTOR and apoptosis pathways *in vivo* and *in vitro*.

## Discussion

The findings of this study demonstrated that ALS attenuated ISO- and Ang II-induced cardiac dysfunction by inhibiting mTOR and apoptosis pathways. Previous studies showed that the effective dose of ALS ranges from 10 to 100 mg/kg per day and has a beneficial effect on cardiovascular diseases, including cardiac hypertrophy, in a rat model (14,24).

In Ang II-induced H9C2 cardiomyocyte hypertrophy, the fold change of BNP mRNA (∼13-fold) was markedly higher compared with that of ANP (∼10-fold) (25). Moreover, the fold change of ANP and BNP was ∼2.0 and 1.2, respectively, after exposure to ISO; these results are comparable to the control group (26). More importantly, ISO induced upregulation of ANP, but not of BNP, in the LVs of the rats (27). In the present study, the change in ANP and BNP was 3.1- and 1.7-fold, respectively, in Ang II-treated H9C2 cells. The difference between ANP and BNP regarding the response to Ang II may be associated with systematic errors, including RNA extraction method, PCR cycle number, etc.

mTOR signaling is considered to play a key role in the growth, proliferation, and survival of various cells (28,29). It was previously suggested that RAP can suppress apoptosis in tunicamycin-treated renal proximal tubular cells through inhibition of mTOR (30). Moreover, mTOR signaling can regulate the survival of cardiomyocytes, whereas mTOR inhibitors induce autophagy and inhibit cardiomyocyte death through activating the adenosine monophosphate-activated protein kinase (AMPK) pathway and inhibiting mTOR signaling (31). Intriguingly, mTOR signaling also plays a potential role in cardiac hypertrophy and fibrosis progression (32). Our findings revealed that both ALS and RAP played similar roles in Ang II-induced cardiac hypertrophy by neutralizing p-mTOR expression. Thus, inactivation of mTOR is an important protective mechanism underlying the involvement of ALS in the pathological process of cardiac hypertrophy. It was previously reported that the activation of RAS stimulates the mTOR pathway in HIV-associated nephropathy (33). In addition, ALS and valsartan were found to improve metabolic signaling, oxidative stress, and myocardial tissue remodeling, at least partially, through the activation of p-mTOR expression (24). These results, together with our findings, suggest that ALS, as a RAS inhibitor, may exert RAP-like effects on ISO- and Ang II-induced cardiac hypertrophy.

Regarding the mechanisms underlying the protective role of ALS in the prevention of ISO- and Ang II-induced cardiac hypertrophy, the other possible explanation is that ALS may inhibit the apoptosis of cardiomyocytes. Delaying cardiomyocyte loss may be a promising therapeutic option after injury, as cardiomyocytes are terminally differentiated and have little potential for mitosis (34). Westermann et al. (35) demonstrated that the number of apoptotic cardiomyocytes was reduced by ALS after myocardial infarction. Zhang et al. (36) also reported that ALS attenuates myocardial cell apoptosis in aged spontaneously hypertensive rats, accompanied by upregulation of Bcl-2 and survivin and downregulation of Bax and caspase-3. Moreover, a recent study highlighted that ALS alleviates cardiac injury and decreases the levels of apoptosis caused by oxygen-glucose deprivation in mice (34). The present study demonstrated that ALS increased H9C2 cardiomyocyte viability *in vitro* under Ang II treatment. Our findings also revealed that ALS significantly attenuated Bax and caspase-3 expression and increased Bcl-2 expression *in vivo* and *in vitro*. These findings further support that ALS played a protective role against ISO- and Ang II-induced H9C2 cardiomyocyte injury.

We need to make clear the limitations in our study. First, mTOR signaling, as the direct regulator of autophagy, has been implicated in ISO- or Ang II-induced pathological cardiac hypertrophy (7,37). Autophagy is usually activated and attenuates LV remodeling and cardiomyocyte apoptosis under pathological conditions, accompanied by activation of AMPK and inhibition of mTOR signaling (38), while the effects of ALS on autophagy, as a cascade signaling of mTOR, in the process of ISO- and angiotensin II-induced cardiac hypertrophy, were not explored. In addition, the anti-fibrotic role of ALS and its underlying mechanism have not been fully elucidated. Furthermore, the effect of ALS on ISO-induced cardiac hypertrophy *in vitro* remains unclear.

The novel findings presented herein were that ALS attenuated ISO- and Ang II-induced cardiac hypertrophy *in vivo* and *in vitro*. It was also demonstrated that ALS treatment suppressed the mTOR and apoptosis pathways in the pathological process of cardiac hypertrophy. These results indicated a novel and direct mechanism by which ALS may be employed in the prevention and treatment of cardiac hypertrophy.
