# **Erratum notice for:** “Aliskiren attenuates cardiac dysfunction by modulation of the mTOR and apoptosis pathways” [Braz J Med Biol Res 2020;53(2): e8793]

**DOI:** 10.1590/1414-431X2023e8793erratum

**Published:** 2023-03-17

Zhengbo Zhao^1*^ https://orcid.org/0000-0003-2613-2397, Han Liu^2*^ https://orcid.org/0000-0002-0477-1537, and Dongmei Guo^3^ https://orcid.org/0000-0002-7332-380X

^1^Department of Cardiovascular Medicine, Jiulongpo District People's Hospital, Chongqing, China

^2^Department of Neurology, Jiulongpo District People's Hospital, Chongqing, China

^3^Department of Cardiovascular Medicine, Nanchuan District People's Hospital, Chongqing, China

Correspondence: Dongmei Guo: <guodongmei677@163.com>

*These authors contributed equally to this study.

The authors notified the Editors of the Brazilian Journal of Medical and Biological Research that there was an error in Figure 1A, due to inappropriate image processing. The conclusion of the results remains unchanged.

The correct Figure 1 is:

**Figure 1:**
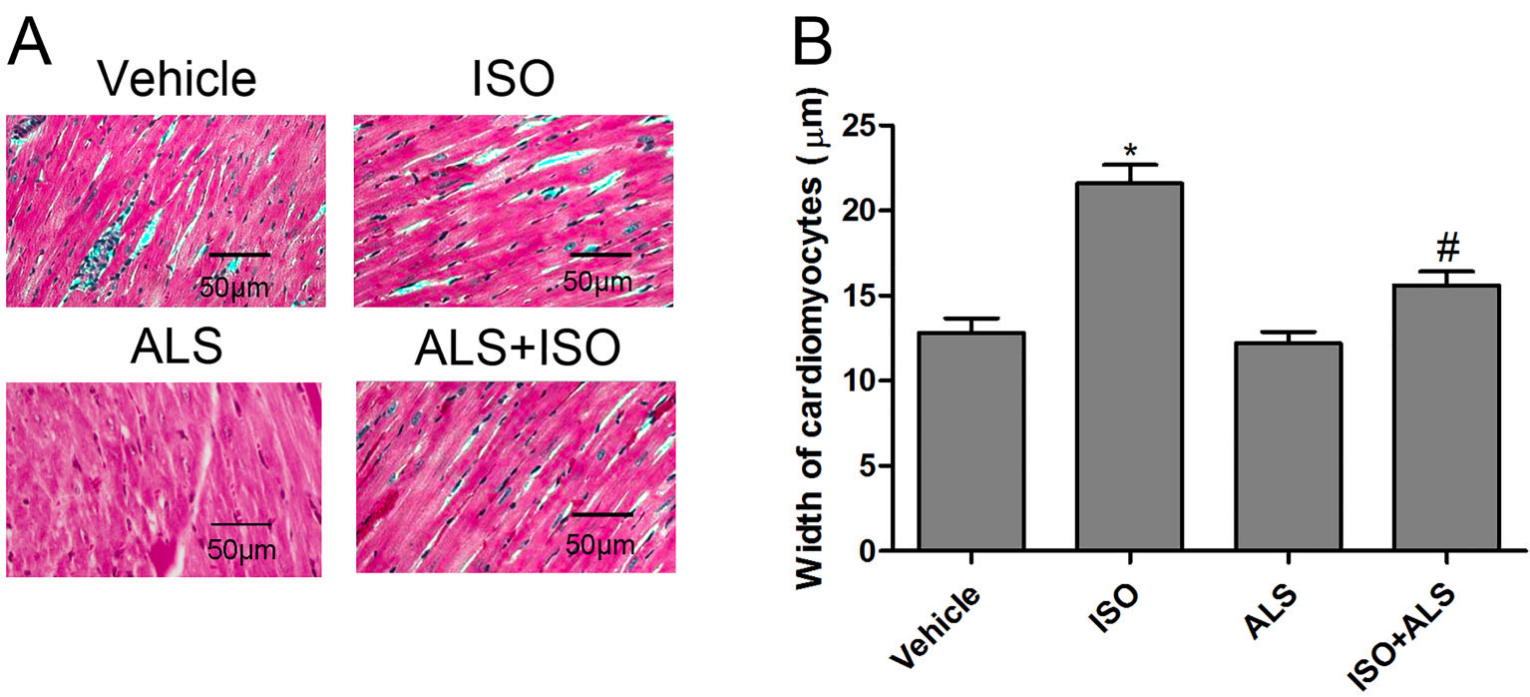
Effect of aliskiren (ALS) on cardiomyocyte size in isoproterenol (ISO)-treated rats. **A**, Hematoxylin and eosin staining was performed and (**B**) the width of cardiomyocytes was measured (n=6 per group). Data are reported as means±SE. *P<0.05 compared with vehicle; ^#^P<0.05 compared with the ISO-treated group (ANOVA followed by Tukey’s post *hoc* test).

